# SHOT ON THE SPOT VACCINATION COMMUNITY OUTREACH

**DOI:** 10.1093/geroni/igac059.1805

**Published:** 2022-12-20

**Authors:** Alissa McIntyre, Rebecca Allen, Brian Cox, Loretta Wilson, Pamela Payne-Foster, JoAnn S Oliver, Yan Luo, Hee Lee

**Affiliations:** University of Alabama, Tuscaloosa, Alabama, United States; The University of Alabama, TUSCALOOSA, Alabama, United States; Alabama Research Institute of Aging, Tuscaloosa, Alabama, United States; Rural Alabama Prevention Center, Eutaw, Alabama, United States; Rural Health Institute/Community Rural Medicine, Tuscaloosa, Alabama, United States; Capstone College of Nursing, Tuscaloosa, Alabama, United States; The University of Alabama, Tuscaloosa, Alabama, United States; University of Alabama, Birmingham, Alabama, United States

## Abstract

Due to the COVID-19 pandemic disproportionately affecting Black communities, The University of Alabama (UA) partnered with the Rural Alabama Prevention Center (RAPC), a community-based healthcare organization to improve the vaccination rate from 34% vaccinated Alabama Black Belt residents to 70% over a year. Health literacy training is provided to community health workers and students who, along with the team members, volunteer at pop-up Shot on the Spot vaccination sites to administer surveys collecting demographics and vaccine hesitancy data. Team members provide health literacy information and answer questions non-vaccinated individuals have. This vaccination intervention has led to drastic rate increases, such as, Choctaw County having a 36.6% increase since the beginning of the project in August (30.7% to 67.30%). However, some counties have low vaccination rate changes, such as, Crenshaw County with a rate change of 15.4% (19.5% to 34.90%). Notably, the Alabama Black Belt currently stands at a higher vaccination rate compared to Tuscaloosa County (UA’s location), only having a rate of 44.3%. Within one year, there have been a total of 44 administered first and second vaccine doses and 435 booster doses, resulting in 50.24% vaccinated Black Belt residents. As the virus evolved into different variants, team members were able to observe an increase in administered booster doses in congruence with the rise of a new coronavirus variant. The partnership formed between RAPC and UA scientists and students is an important step in improving vaccination rates and building community research on minority and diverse populations.

